# Rinsability of Orthophthalaldehyde from Endoscopes

**DOI:** 10.1155/2012/853781

**Published:** 2012-05-16

**Authors:** Norman Miner, Valerie Harris, Natalie Lukomski, Towanda Ebron

**Affiliations:** MicroChem Laboratory, Inc., 1107-C South Airport Circle, Euless, TX 76040, USA

## Abstract

Orthophthalaldehyde high level disinfectants are contraindicated for use with urological instruments such as cystoscopes due to anaphylaxis-like allergic reactions during surveillance of bladder cancer patients. Allergic reactions and mucosal injuries have also been reported following colonoscopy, laryngoscopy, and transesophageal echocardiography with devices disinfected using orthophthalaldehyde. Possibly these endoscopes were not adequately rinsed after disinfection by orthophthalaldehyde. We examined this possibility by means of a zone-of-inhibition test, and also a test to extract residues of orthophthalaldehyde with acetonitrile, from sections of endoscope insertion tube materials, to measure the presence of alkaline glutaraldehyde, or glutaraldehyde plus 20% w/w isopropanol, or ortho-phthalaldehyde that remained on the endoscope materials after exposure to these disinfectants followed by a series of rinses in water, or by aeration overnight. Zones of any size indicated the disinfectant had not been rinsed away from the endoscope material. There were no zones of inhibition surrounding endoscope materials soaked in glutaraldehyde or glutaraldehyde plus isopropanol after three serial water rinses according to manufacturers' rinsing directions. The endoscope material soaked in orthophthalaldehyde produced zones of inhibition even after fifteen serial rinses with water. Orthophthalaldehyde was extracted from the rinsed endoscope material by acetonitrile. These data, and other information, indicate that the high level disinfectant orthophthalaldehyde, also known as 1,2-benzene dialdehyde, cannot be rinsed away from flexible endoscope material with any practical number of rinses with water, or by drying overnight.

## 1. Introduction

There are many references in the scientific literature noting anaphylactic shock reactions for patients examined with cystoscopes [1–3], laryngoscopes [4], and colonoscopes [5] disinfected with orthophthalaldehyde (OPA). Serious mucosal injury to the lips, tongue, throat, and esophagus have been reported for patients due to transesophageal echocardiography (TEE) probes that had been high level disinfected with OPA [6, 7]. Many of the case reports deal with the clinical condition of the patient after exposure to endoscopes or TEE probes disinfected with OPA and detailed procedures for rinsing the equipment are limited to “copious.” This description of the rinsing procedure left open the possibility that the endoscopes were not thoroughly rinsed free of OPA. However, several of the publications are more specific identifying three sequential one-minute immersions in 2 gallons of water [6], or four sequential immersions each in 2 liters of water [7]. The manufacturer's instructions for manual rinsing are to immerse the medical device in 2 gallons of water and to leave the device immersed for 1 minute and repeat this procedure three times with fresh water each time. There are special instructions on the OPA package insert for TEE probes that include enclosing the TEE probe in a sterile protective sheath when performing TEE. These instructions suggest that a residue of OPA might remain on the TEE probe even after careful rinsing, and the protective sheath is used to provide a physical barrier to protect the patient from any residue of OPA. The instructions for use for OPA high level disinfectant solution carry a contraindication warning that OPA should not be used to process any urological instruments used to examine patients with bladder cancer. The bladders of these patients are examined frequently with cystoscopes, suggesting that the endoscope might initially carry OPA into the tissues of the urethra to sensitize the patient, and the allergic reaction occurs with reexamination due to continuing residues of OPA on the cystoscope.

While there are label warnings against the use of OPA high level disinfectants for the disinfection of cystoscopes, and special instructions for ensheathing TEE probes, there is no warning or contraindication for the use of OPA to disinfect laryngoscopes or colonoscopes, for which there are peer-reviewed reports of serious allergic reactions. The materials of insertion tubes for all manufacturers of cystoscopes, laryngoscopes, colonoscopes, bronchoscopes, and gastroscopes, are all constructed using the same or similar flexible polymeric material. However, there is no contraindication on the OPA labels that it should not be used to disinfect laryngoscopes, bronchoscopes, gastroscopes, or colonoscopes, which might be taken to imply that these other medical devices can be adequately rinsed to remove the OPA residues, while cystoscopes and TEE probes cannot. The investigations of this paper were carried out to determine how many rinses are necessary to remove OPA from endoscope insertion tube materials, and the results indicated surprisingly that OPA adsorbs to the endoscope materials and cannot be rinsed away with any practical or even greatly excessive numbers of rinses with water. The test methods and results of this paper are the first to identify by observed zones of inhibition and chemical extraction that OPA adheres or adsorbs to the materials of endoscopes. The adverse clinical reactions reported from endoscopes and TEE probes disinfected with OPA are not the result of poor or insufficient rinsing procedures. If residues of OPA remain on endoscopes, cannot be rinsed away with any practical number of rinses, and can be transferred to patients, then some consideration should be given to the nature and potential toxicity of the OPA chemical, which is 1, 2-benzene dialdehyde, that is, a modified benzene compound. Possibly the warnings regarding OPA should be extended to other medical devices that remain in place for a length of time sufficient to produce mucosal injury, or might be used for examinations and reexaminations of the esophagus, stomach, colon, bronchioles, and lungs; or possibly some OPA removal technique such as rinsing in an organic solvent should be developed.

## 2. Materials and Methods

Endoscope insertion tube parts representing many models of flexible endoscopes were obtained new from Endoscope Repair, Inc., and Olympus America, Inc. Endoscope insertion tubes (colonoscope part no. 417900, Olympus America Inc., and Pentax-compatible part no. EG-2900/2901), and endoscope bending rubbers made of Viton, silicone, and polyvinylchloride (obtained from Endoscope Repair, Inc.) and bending rubber “A” (obtained from Olympus America Inc.), all of which are parts of endoscopes that contact disinfectants and the patient, were stripped of any metal parts, and then cut into approximately 2.0 cm × 1.0 cm sections. These sections of endoscope materials were soaked in 2.4% w/w alkaline glutaraldehyde Solution (GA) (Cidex Activated Dialdehyde Solution, Advanced Sterilization Products, Irvine, CA); 3.4% w/w GA plus 20% w/w isopropanol Solution (GA-IPA) (Aldahol High Level Disinfectant, Healthpoint, LTD, Fort Worth, TX); or 0.55% w/w orthophthalaldehyde Solution (OPA) (Cidex OPA, Advanced Sterilization Products, Irvine, CA) for 10.0 min at 20°C for GA-IPA, and 25°C for GA, and OPA, the label temperature directions for these disinfectants. The endoscope insertion tube materials were then rinsed multiple times in 100 mL of filtered tap water per rinse. The pieces were soaked and agitated in each rinse for 1.0 min, and then moved from rinse to rinse with sterile stainless steel forceps. These rinses for 2.0 cm^2^ sections of endoscope insertion tube materials were proportionally similar in volume and rinse time to the rinses directed by the disinfectant manufacturers for whole endoscopes.

To determine how much disinfectant might remain on a complete endoscope before rinsing, an intact Olympus gastroscope was immersed in GA-IPA for 10.0 min, removed from the disinfectant, and the interior channels were then drained and purged with air. The endoscope was weighed before and after exposure to the disinfectant. About 20.0 g (20.0 mL) of disinfectant remained on a gastroscope as ready to be rinsed. The small sections of endoscope material were similarly weighed before and after exposure to the disinfectants to determine that about 0.2 g (mL) of disinfectant remained on these sections. This information was used to calculate the total dilution factor following three two-gallon rinses of an endoscope, or various numbers of rinses with 100 mL of water for the small sections.

A culture of *Staphylococcus aureus, *American Type Culture Collection no. 6538, was spread over the surface of trypticase soy agar (Becton Dickinson) in a 100 × 15 mm plastic petri dish (Fox Scientific). The disinfected and rinsed 2.0 cm × 1.0 cm pieces of endoscope materials were placed individually onto the surface of the bacteria-seeded agar in petri plates, and the plates were incubated for 48 ± 8 hrs at 35 ± 2°C to form a confluent “lawn” of bacteria. Zones of inhibition (ZOI) where the bacteria could not grow were measured around the endoscope materials in mm from side to side. These ZOI represented inhibitory concentrations of the high level disinfectants if they had not been rinsed away, and the disinfectants then leached into the agar from the sections of processed and rinsed endoscope materials. As positive controls, sections of the endoscope materials were soaked in the disinfectants as described above, not rinsed, and then placed onto the trypticase soy agar surfaces with bacterial lawns. After incubation, each of the three types of disinfectants gave large ZOI indicating the disinfectants could leach away from the endoscope materials and inhibit the growth of the *S. aureus.* As negative controls, the sections of the endoscope materials were soaked in water, without exposure to any disinfectant, and then placed onto the agar with bacterial lawns, and the result determined that there were no chemicals in the insertion tube materials able to give a ZOI in these tests. Three sections of endoscope materials were tested each time.

Variations of this test using Viton bending rubber (the most commonly used bending rubber) and insertion tube material included increasing the numbers of rinses up to fifteen 100 mL rinses, multiple exposures to the test disinfectants up to five 10.0 min exposures per day, various post rinse drying times, and tests of the effects of a final wipe with isopropanol.

As an additional method to confirm that the ZOI were the result of OPA, a 2.0 cm × 1.0 cm section of Olympus colonoscope insertion tube was soaked in OPA for 10.0 min at ambient temperature, followed by three rinses in 100 mL of water per rinse. The soaked and rinsed section was then placed into 5.0 mL of acetonitrile (Sigma-Aldrich) in a 20 mL glass vial and agitated on a vortex mixer for 1.0 min to extract the OPA. This procedure was repeated with an identical section of insertion tube that had been rinsed with water and not soaked in OPA. The insertion tube sections were removed from the vials, and acetonitrile extractions, along with a sample of OPA were analyzed in a high performance liquid chromatography (HPLC) machine. The HPLC analysis of acetonitrile from the control section not soaked in OPA produced no signal on the chromatogram, while the analysis of the section soaked in OPA gave the same signal as OPA itself.

## 3. Results

Table 1 measures the ZOI surrounding four types of bending rubber, and an endoscope insertion tube following a 10.0 min exposure to GA, GA-IPA, or OPA, followed by three, seven, or fifteen rinses each for 1.0 min with 100 mL of water. There were no ZOI surrounding the endoscope materials that were soaked in GA or GA-IPA, indicating that these disinfectants had been rinsed away with three or seven rinses each in 100 mL of fresh water. With the exception of bending rubber A, there were consistent ZOI around all of the endoscope materials exposed to OPA, even after three, seven, or fifteen serial rinses each in 100 mL of fresh water. There was about a 20% reduction in the size of the ZOI for OPA after 15 serial rinses as compared to three rinses.

Table 2 measures the buildup of the high level disinfectants on the insertion tube materials after one 10 min exposure to the disinfectants, followed by five 10 min exposures to GA, GA-IPA, or OPA. Each exposure was followed by three rinses each in 100 mL of water before placing the insertion tube material onto the surface of the petri plates spread with *S. aureus*. There was no buildup following these multiple exposures to GA or GA-IPA. There was a 50% increase in the zone sizes following five exposures to OPA for the endoscope insertion tube material, but not for the Viton bending rubber.

Table 3 measures the effects on the zones of inhibition of exposing the endoscope materials to OPA for one 10 min exposure, rinsing three times, followed by drying the materials at ambient temperature for 1.0, 2.0, 6.0 hrs, and overnight. This test measures the potential for the OPA to evaporate away from the endoscope material. Tests were repeated three times. There was a ZOI immediately after the exposure of the Viton bending rubber to OPA, and this dissipated after drying for 2.0 hrs. The ZOI remained on the colonoscope insertion tube material for up to 6.0 hrs, and then this single exposure evaporated after drying overnight.

Table 4 simulates the clinical frequency of using an endoscope about five times per day, with high level disinfection and rinses in between each use, and repeating this use pattern for a second day. Zones of inhibition of *S. aureus *were measured for 2 cm × 1 cm sections of Viton bending rubber and endoscope insertion tube material after they were each soaked five times in OPA for 10.0 min, followed by three 100 mL rinses of water between each disinfectant exposure, and overnight drying. This procedure was repeated for a second day. There were no zones of inhibition for the Viton bending rubber. Previous tests as shown in Table 3 above indicate that the overnight drying allowed the OPA to evaporate from the Viton sections, but large zones with an average of 22 mm after one day, and an average of 27 mm after the second day surrounded the colonoscope insertion tube sections. The Viton result is immaterial because the bending rubber sections are always attached to the insertion tube material.

A test was done to try to remove OPA from the insertion tube material by wiping the insertion tube with a cotton ball soaked in 75% isopropanol, and then drying for 15 min, or wiping the insertion tube material with 99.9% acetonitrile or 3% hydrogen peroxide (solvents for OPA). There was no reduction in the size of the ZOI following a wipe with isopropanol. Acetonitrile was able to wipe the OPA away from the Viton endoscope material, but not from the insertion tube material. Hydrogen peroxide was not able to remove the OPA from either the Viton material or the insertion tube material. There were no zones of inhibition around the Viton or the insertion tube material wiped with acetonitrile or hydrogen peroxide. These results are shown in Table 5.

**Figure 1:**
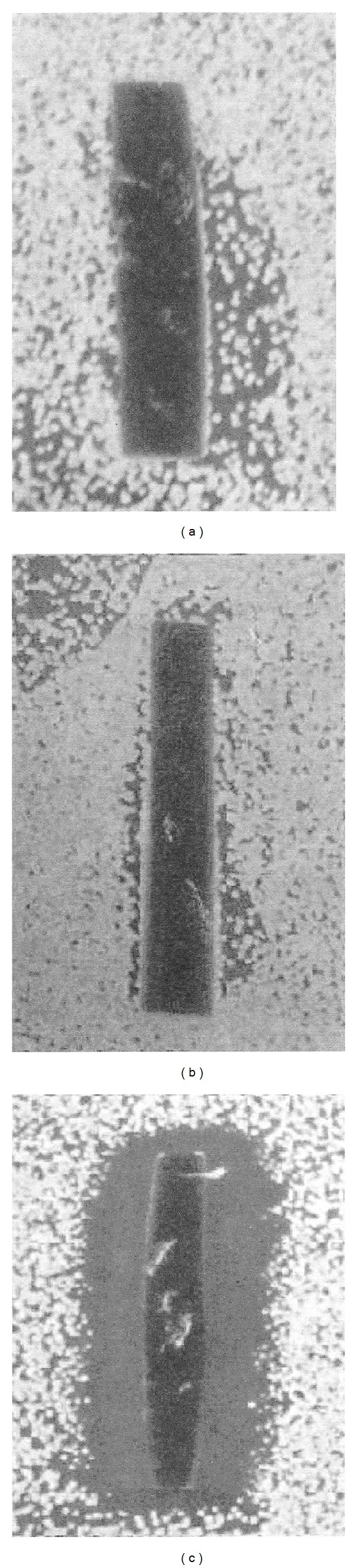
Typical zone of inhibition or no zone of inhibition on agar surfaces with a lawn of *Staphylococcus aureus* surrounding 2 cm × 1 cm sections of Viton endoscope bending rubber exposed from left to right to GA-IPA, GA, or OPA for 10.0 minutes, and then rinsed three times each with 100 mL of water. These positive control sections of the endoscope materials were then placed onto the trypticase soy agar surfaces with bacterial lawns. Negative control sections of the endoscope materials were soaked in water, without exposure to any disinfectant, and then placed onto the agar with bacterial lawns, and the result determined that there were no chemicals in the insertion tube materials able to give a ZOI in these tests (not shown).

Figure 1 is a photograph of the zones of inhibition surrounding 2 cm × 1 cm sections of Viton bending rubber as soaked for 10 min in (a) GA-IPA, (b) GA, and (c) OPA, and then rinsed three times each in 100 mL water. There were no ZOI surrounding the Viton bending rubber soaked in GA-IPA or GA, indicating those disinfectants have been rinsed away. The Viton bending rubber soaked in OPA gave a large ZOI, indicating it could not be rinsed away.

## 4. Discussion

There have been many reports in the scientific literature, some as referenced in this paper, of mucosal injuries and serious allergic reactions of patients treated with endoscopes disinfected with OPA and rinsed with water. The question is are these events happening because the endoscopes and TEE probes are not being adequately rinsed with water, or for some other reason such as the OPA adsorbs to the polymeric material of transesophageal echo probes and the insertion tubes of flexible endoscopes and cannot be rinsed away? Orthophthalaldehyde, glutaraldehyde, and glutaraldehyde antimicrobially enhanced by combination with isopropanol [8] are all toxic allergenic biocidal chemicals. However, this is of little concern if the OPA, GA, and GA-IPA can be rinsed away from the endoscopes and disposed of safely. The zone-of-inhibition tests do not try to measure concentrations remaining on endoscopic material. The tests simply identify if the disinfectant can remain adsorbed to the endoscope material or not. These tests indicate that GA high level disinfectants and high level disinfectants composed of GA plus isopropanol do rinse away from the endoscope materials with three copious rinses of water, while OPA does not rinse away from endoscope materials.

The rinsing directions for Cidex OPA are to rinse three times in 2.0 gal of water (7570 mL) per rinse. Assuming the endoscopes carry a residue of about 20 mL of disinfectant after soaking in a disinfectant, measured as described above in the methods section, the dilution factor is 378 for one rinse (7570 mL ÷ 20 mL), or about 54 × 10^6^ for three rinses.

 The small sections tested each carry about 0.2 mL of disinfectant solution. When rinsed in 100 mL of water, that is a 500-fold dilution of the disinfectant per rinse. Three such rinses give a dilution factor of 125 × 10^6^, which is similar or in excess of rinsing an entire endoscope in two gallons of water three times. Seven rinses of the small sections of insertion tube material in 100 mL of water per rinse (100 mL ÷ 0.2 mL) is a dilution factor of about 7.5 × 10^18^, and fifteen rinses is about 3.05 × 10^40^, a huge dilution factor. Even after seven or fifteen serial rinses these insertion tube materials soaked in OPA, with the exception of bending rubber “A,” gave zones of inhibition indicating there was still OPA on these insertion tubes and bending rubber materials. The OPA is not rinsing away from the endoscope insertion tube materials. The OPA is building up on the materials with each successive exposure, and the OPA is not evaporating from the materials in any practical drying time relative to clinical demands to reuse the disinfected endoscopes several times per day, or even overnight.

In terms of rinsability, all of the chemicals of the three high level disinfectants tested reacted as would be expected from the physical characteristics of their solubility in water and their vapor pressures (ability to evaporate). Both GA and IPA are very soluble in water, infinitely so with IPA solubility in water, and 64 g of GA are soluble in 100 mL of water. These two chemicals consistently rinsed away from the endoscope materials with three rinses in water and did not build up on the endoscope material surfaces after repeated exposure and rinses.

OPA has a very low solubility in water at 0.60 g per 100 mL of water, and the solubility of OPA in alcohol is not much greater at 11 g/100 mL of alcohol (not specified). The endoscope surfaces exposed to OPA and wiped with isopropanol still gave zones of inhibition in these tests. GA and IPA have high vapor pressures and would be expected to slowly evaporate away from the endoscope surfaces even if they were not rinsed away with water. OPA has a very low vapor pressure of 0.0052 mm Hg at 21°C, which explains why it has a more tolerable odor than GA, and also explains why it does not evaporate from the endoscope surfaces drying overnight.

The standard method to measure that disinfectants can be rinsed away from medical equipment is to rinse the equipment in water and then measure the water for the presence of the disinfectant. After some series of rinses, including overnight extractions, the concentration of the disinfectant in the rinse water should be below any known levels of toxicity. However, if the disinfectant adsorbs to the medical instrument, then the disinfectant, or some concentration of the disinfectant, will not be in the rinse water to be measured. That is what we think is happening with measurements for residues of OPA. The manufacturer, regulatory agencies, and clinicians might not expect or look for such a scenario where the OPA is adsorbed to the endoscope material and is not dissolved in the rinse water, and thus not detectable in rinse water by ordinary methods.

We believe the body of evidence as listed here indicates that OPA adsorbs to the polymeric materials of endoscopes and other medical devices and cannot be rinsed away. (1) There are numerous reports in the scientific literature of mucosal injuries, and allergic reactions in patients exposed to equipment disinfected with OPA (cited in the introduction), (2) TEE probes, as disinfected with OPA and rinsed, must be placed into a protective cover before used with patients. (3) Animal toxicity studies [9, 10] indicate that OPA is a potent allergen. It is reasonable to assume that for every case of a mucosal injury or anaphylactic shock reaction that is published, many more are not published. (4) The contraindication for the use of OPA to disinfect cystoscopes, and the special directions for TEE probes is published on the product label by the manufacturers. (5) The materials of endoscope insertion tubes are all similar to the materials for cystoscope insertion tubes for which there are numerous reports of serious allergic reactions in the scientific literature. (6) Considerations of the low water solubility of OPA and its low vapor pressure further suggest that OPA might not be expected to be rinsed or evaporated away from endoscopes, and finally, (7) the data of this paper demonstrate that OPA cannot be rinsed away from most endoscope insertion tube materials even with very great numbers of rinses, dilutions, and drying times.

What is different about cystoscopes from other endoscopes such as bronchoscopes, gastroscopes, colonoscopes, and laryngoscopes? The flexible polymeric construction materials of all of these endoscopes are similar. Cystoscopes as used to monitor bladder cancer are introduced into the body more frequently than other endoscopes, possibly sensitizing the body with the first introduction of OPA residues, and the body reacts to the allergen with additional examinations.

We encourage other scientists to repeat and expand on our studies of the rinsability of OPA. Possibly insertion tubes can be ensheathed to prevent contact of the mucous membranes with OPA disinfected insertion tubes in the same manner as with TEE probes Possibly some system of rinsing the endoscopes with such organic solvents as acetonitrile or hydrogen peroxide [7] to remove the OPA could be developed, although these solvents did not remove the OPA in tests reported here. Automatic endoscope reprocessing machines might or might not rinse in some manner more effectively than manual rinses. These are expensive machines, not common to a research laboratory. This needs to be studied. Possibly endoscopes might be made of polymeric material such as bending rubber “A” that has been tested and is known to not adsorb high level disinfectants of any type. Clinicians should be made aware that any endoscope disinfected with OPA might induce an allergic reaction or mucosal injury, regardless of thorough rinsing procedures. Thus the answer to the question that began this study is that OPA cannot be rinsed away from endoscopes by serial rinses in water, and those persons responsible for reprocessing endoscopes should know that allergic reactions of patients treated with endoscopes disinfected with OPA are not likely due to inadequate rinsing of the endoscopes with water.

##  Conflict of Interests and Source of Funding

Norman Miner discloses that he is the inventor of Aldahol High Level Disinfectant, with a royalty interest. The studies of this paper were funded entirely by MicroChem Laboratory.

## Figures and Tables

**Table 1:** Zones of Inhibition (ZOI) of *S. aureus *on four types of bending rubbers and an endoscope insertion tube section following exposure to GA-IPA at 20°C, GA and OPA at 25°C, and rinses with 100 mL of water. The numbers reported are the mean ± standard deviation of three replicates in mm.

Endoscope material	GA @ 25°C or GA-IPA @ 20°C	OPA @ 25°C		
	Three or seven rinses ZOI (mm)	Three rinses ZOI (mm)	Seven rinses ZOI (mm)	Fifteen rinses ZOI (mm)
Viton	0 ± 0	10 ± 0.6	11 ± 0	8 ± 3.5
Silicone	0 ± 0	18 ± 0.6	18 ± 1.2	
PVC	0 ± 0	18 ± 0.6	20 ± 0.6	
Rubber A	0 ± 0	0 ± 0	0 ± 0	
Insertion tube	0 ± 0	17 ± 1	18 ± 1.5	14 ± 1.2

**Table 2:** Zones of Inhibition (ZOI) of *S. aureus *on endoscope insertion tube material and Viton bending rubber following one or five 10 minute exposures to the high level disinfectant, followed by three 100 mL rinses after each exposure. The numbers reported are the mean ± standard deviation of three replicates in mm.

Endoscope material	GA @ 25°C or GA-IPA @ 20°C		OPA @25°C	
	One 10 min exposure ZOI (mm)	Five 10 min exposures ZOI (mm)	One 10 min exposure ZOI (mm)	Five 10 min exposures ZOI (mm)
Viton bending rubber	0 ± 0	0 ± 0	12 ± 0.6	17 ± 1

Colonoscope insertion tube	0 ± 0	0 ± 0	16 ± 0	24 ± 1

**Table 3:** Zones of Inhibition (ZOI) of *S. aureus* on Viton bending rubber and endoscope insertion tubes after exposure to OPA for 10 minutes, followed by three 100 mL rinses and various drying times. The numbers reported are the mean ± standard deviation of three replicates in mm.

Drying times	OPA @ 25°C	
	Viton bending rubber ZOI (mm)	Colonoscope insertion tube ZOI (mm)
Not dried	8 ± 0	
1.0 hr	4 ± 3.5	
2.0 hrs	0 ± 0	13 ± 1
6.0 hrs		12 ± 3.2
Overnight		0 ± 0

**Table 4:** Zones of Inhibition (ZOI) of *S. aureus *for sections of Viton bending rubber and endoscope insertion tube sections following five 10.0 minute exposures to OPA at 25°C for one day, three 100 mL rinses with water, and overnight drying, and then repeating the exposure, rinsing, and overnight drying for a 2nd day. The numbers reported are the mean ± standard deviation of three replicates in mm.

Endoscope material	OPA @ 25°C	
	1 day	2 days
	5 exposures, 3 water rinses, and overnight drying ZOI (mm)	5 exposures, 3 water rinses, and overnight drying ZOI (mm)
Viton	0 ± 0	0 ± 0
Insertion tube	22 ± 1	27 ± 2.1

**Table 5:** Zones of Inhibition (ZOI) of the growth of *S. aureus *surrounding sections of endoscope insertion tube materials after three 10-minute exposures to OPA, three 100 mL rinses, followed by wiping with acetonitrile or 3% hydrogen peroxide and three 100 mL rinses. The numbers reported are the mean ± standard deviation of three replicates in mm.

Endoscope material	OPA	Acetonitrile		3% hydrogen peroxide	
	Three 10 min exposures/rinses^a^ ZOI (mm)	Three 10 min OPA exposures/rinses^a^, Wipe/rinses^b^ ZOI (mm)	Wipe/ rinses^b^ ZOI (mm)	Three 10 min OPA exposures/rinses^a^, Wipe/rinses^b^ ZOI (mm)	Wipe/ rinses^b^ ZOI (mm)
Viton	14 ± 0	0 ± 0	0 ± 0	11 ± 1	0 ± 0
Insertion tube	21 ± 1	19 ± 0.6	0 ± 0	20 ± 1	0 ± 0

^a^ Inbetween each 10 minutes OPA soak materials were rinsed three times with 100 mL of tap water.

^b^ After wiping with acetonitrile or hydrogen peroxide materials were rinsed three times with 100 mL of tap water.
